# Letter from Paris

**Published:** 1868-07

**Authors:** D'Oyley Evans


### ARTICLE VIII.

### *Letter from Paris.*

Dear Doctor:

As promised, I send you an account of a case of smallpox treated with a favorite medicine of mine carbolic acid; by Horatio Yates M. D. of *Kingston General Hospital*, Canada. It seems "like carrying coals to Newcastle," to send such information to you from here, when the cure was effected so near "home," but if the intelligence has already reached you, simply throw the letter aside without any formality, if not and you think it worthy of attention please find room for it in your excellent Journal—and I should be glad if some of our *Medical* brethren would try the local treatment when opportunity offers and report the result, for in future much disfigurement may be prevented after that terrible scourge, which fills every mind with horror and apprehension when it visits us.

The following is a letter of Dr. Yates.

"A beautiful young lady, in passing with her friends through Kingston, was seized with the primary fever of variola. From the violence of the symptoms, the intense head-ache followed by delirium, the vomiting, the atrocious spinal pains— an aggravated case was apprehended. The eruption duly appeared, and she was at once removed from the Hotel to a small-pox ward in the Watkins wing of the Kingston Hospital. The whole body was very thickly studded with the eruption, and many patches of confluence appeared on the face and forehead. The mouth, fauces, and conjunctivæ were covered with the eruption.

The first object, after saving the patients life, was to prevent disfigurement. The ward of which she and the nurse were sole occupants is large, lofty and thoroughly ventilated. The temperature throughout was mantained at 64° during the day and 58° at night. The room was darkened and a candle only used for light.

But I am induced to transcribe as briefly as possible the local treatment, its success having been so gratifying. We have for years expected to prevent pitting of the face by excluding light and air from the surface.

For many years I have habitually used nitrate of silver, tincture of iodine, mercurial ointmeut, &c., to the face for this object, with variable success.

In this case I employed an ointment of carbolic acid, two drachms; mutton suet two ounces, and coloured with lamp-black. This ointment was spread thickly upon black cotton wadding, which was applied over the face and forehead, holes being cut for the eyes, nostrils and mouth. This mask was changed every second day and the face gently washed with soap and warm water and then, including the whole body, with warm water impregnated with carbolic acid.

From the commencement of the eruption no unpleasant symptoms occurred. There was none of that intolerable itching, and no secondary fever.

One arm only of the patient was smeared daily with the ointment, the contrast in the character of the pocks on the arms was remarkable. The pustules on the arm operated upon were not nearly as full, and the scales fell off earlier than those of the other arm.

Whatever part the carbolic acid played in the case in preventing pitting and how much the exclusion of air had to do with it I cannot say, but this we know that carbolic acid is a powerful disinfectant, and that it destroys microscopic vegetations and it certainly prevented the peculiar odour of small-pox in the room, and, as I have already said, I attribute the absence of secondary fever to this agent."

Since writing the above I have seen a friend, an eminent Physician of this city, who informed me that he had, since three years applied carbolic acid in cases of small-pox, and has never failed in obtaining perfect success in preventing pitting, the same gentleman Dr. Declat, has been very successful in his treatment of cancerous tumors with carbolic acid spray and has promised to acquaint me with his method, which has gained him so great a reputation, that almost hopeless cases and those persons abandoned by celebrated Physicians in Europe come to him for relief and in the hope of prolonging their existence at least, if not being entirely cured. Dr. Declat is at present occupied in writing and preparing for the press, his experience in cancerous diseases treated with carbolic acid. I hope to be able to carry to you in June when I return home to my native land, the proof sheet if not the work completed, and have no doubt that it will be very interesting and instructive.

Yours truly

D'Oyley Evans.

